# Platelet Reduction after Transcatheter Aortic Valve Implantation: Results from the PORTRAIT Study

**DOI:** 10.3390/jcm13061579

**Published:** 2024-03-10

**Authors:** Federica Jiritano, Michele Di Mauro, Giuseppe Filiberto Serraino, Pasquale Mastroroberto, Elena Caporali, Enrico Ferrari, Mariusz Kowalewski, Roberto Scrofani, Leonardo Patanè, Giuseppe Visicchio, Domenico Paparella, Giosuè Falcetta, Andrea Colli, Matteo Matteucci, Giangiuseppe Cappabianca, Francesco Pollari, Theodor Fischlein, Roberto Lorusso

**Affiliations:** 1 Cardiac Surgery Unit, Department of Experimental and Clinical Medicine, University “Magna Graecia” of Catanzaro, 88100 Catanzaro, Italy; serraino@unicz.it (G.F.S.); mastroroberto@unicz.it (P.M.); 2 Department of Cardio-Thoracic Surgery, Heart & Vascular Centre, Maastricht University Medical Centre (MUMC), Cardiovascular Research Institute Maastricht (CARIM), 6229 Maastricht, The Netherlands; mdimauro1973@gmail.com (M.D.M.); kowalewskimariusz@gazeta.pl (M.K.); matteomatteucci87@gmail.com (M.M.); robertolorussobs@gmail.com (R.L.); 3 Thoracic Research Centre, Collegium Medicum Nicolaus Copernicus University, Innovative Medical Forum, 85-094 Bydgoszcz, Poland; 4 Cardiac Surgery, Istituto Cardiocentro Ticino, 6900 Lugano, Switzerland; dottoressacaporali@gmail.com (E.C.); enrico.ferrari@eoc.ch (E.F.); 5 Clinical Department of Cardiac Surgery, Central Clinical Hospital of the Ministry of Interior and Administration, Centre of Postgraduate Medical Education, 01-813 Warsaw, Poland; 6 Cardiac Surgery Unit, Luigi Sacco Hospital, 20157 Milan, Italy; roberto.scrofani@policlinico.mi.it; 7 Department of Cardiology Cardiac Surgery (Centro Cuore), Centro Clinico Diagnostico G.B. Morgagni, 95125 Catania, Italy; lpatane@centrocuore.it; 8 Department of Cardiac Surgery, Santa Maria Hospital, GVM Care & Research, 70124 Bari, Italy; visicchio@virgilio.it; 9 Dipartimento Scienze Mediche e Chirurgiche, Università di Foggia, 71122 Foggia, Italy; domenico.paparella@unifg.it; 10 University Hospital—Section of Cardiac Surgery, 56124 Pisa, Italy; giosuefalcetta@gmail.com (G.F.); colli.andrea.bcn@gmail.com (A.C.); 11 Department of Surgical and Morphological Sciences, Circolo Hospital, University of Insubria, 21100 Varese, Italy; giangi.cappabianca@googlemail.com; 12 Klinikum Nürnberg, Cardiovascular Center, Paracelsus Medical University, 90419 Nuremberg, Germany; francesco.pollari@klinikum-nuernberg.de (F.P.); theodor.fischlein@klinikum-nuernberg.de (T.F.)

**Keywords:** thrombocytopenia, platelet, transcatheter aortic valve implantation, bioprosthesis, aortic valve

## Abstract

**Background:** An unexplained condition that follows transcatheter aortic valve implantation (TAVI) is platelet count reduction (PR). According to published research, patients with balloon-expandable valves (BEVs) had a greater PR than those with self-expandable valves (SEVs). **Objectives:** The purpose of this study was to investigate the incidence and clinical effects of PR following TAVI. **Methods:** In total, 1.122 adult TAVI patients were enrolled. Propensity score matching was carried out in a 1:1 ratio between patients with BEVs and those with SEVs. The analysis included changes in platelet count, in-hospital mortality, and early postoperative adverse events. **Results:** Notably, 632 patients were matched (BEV:316; SEV:316). All patients’ post-procedural platelet counts changed according to a parabolic curve, using a mixed regression model for repeated analyses (estimate = −0.931; standard error = 0.421; *p* = 0.027). The platelet count varied comparably in patients with BEVs and SEVs (estimate = −4.276, standard error = 4.760, *p* = 0.369). The average time for obtaining the nadir platelet count value was three days after implantation (BEV: 146 (108–181) vs. SEV: 149 (120–186); *p* = 0.142). Overall, 14.6% of patients (92/632) had post-procedural platelet count <100,000/µL. There was no difference between the two prosthesis types (BEV:51/316; SEV:41/316; *p* = 0.266). Thrombocytopenia was found to be significantly linked to blood product transfusions, lengthier stays in the intensive care unit and hospital, and in-hospital mortality. **Conclusions:** TAVI, irrespective of the type of implanted valve, is linked to a significant but temporary PR. Thrombocytopenia increases the risk of serious complications and in-hospital death in TAVI patients. To explore and clarify the causes and associated effects, further prospective research is necessary.

## 1. Introduction

Transcatheter aortic valve implantation (TAVI) completely changed the way we deal with high-risk patients with severe aortic stenosis, becoming a valuable daily therapeutic option. Lately, the role of TAVI has evolved worldwide thanks to the expansion of guideline recommendations to include patients with lower surgical risk [1,2]. TAVI technology improvement and process simplification occurred thanks to new prostheses (e.g., smaller delivery sheets or the ability to reposition) and operator experience, resulting in an improved safety profile and fewer procedure-related adverse events such as stroke, pacemaker implantation, paravalvular leaks, and access site complications [3,4]. 

Among periprocedural TAVI complications, platelet count reduction (PR) has always been neglected, and its incidence and relevance were not well known until the recent interest it raised among researchers [5,6,7]. Post-TAVI PR has been explained by several general mechanisms, such as inflammation, drug toxicity (e.g., heparin, aspirin or other antiplatelet drugs, warfarin, and novel oral anticoagulants), mechanical damage from shear stress (e.g., in the event of a paravalvular leak), the activation of the coagulation cascade, decreased platelet production, impaired platelet renewal, and dilution pseudo-thrombocytopenia. Although the causes of these events remain unclear, theories are based on research involving surgical bioprostheses. While the etiology seemed multifactorial, PR after TAVI is linked to poor clinical outcomes. Dvir and colleagues found that patients with a high reduction in platelet count (≥50%) had a worse 1-year survival rate compared to individuals with a lesser platelet count decline (*p*  < 0.001) (1-year survival: 65.8% vs. 83.9%) [8]. PR was found to be associated with acute kidney failure, vascular complications, bleeding complications, and a high mortality rate [5,6]. Moreover, limited data are available on the occurrence of PR after balloon-expandable valve (BEV) and self-expandable valve (SEV) implantation [5,7]. Only a few studies have demonstrated that the use of BEVs was linked to a higher drop in the post-procedural platelet count than the use of SEVs [5,7].

The aim of this study was to analyze the platelet count variation after TAVI and the prognostic implications for the early clinical outcomes related to this phenomenon.

## 2. Materials and Methods

The Post-Operative Thrombocytopenia After Bio-Prosthesis Implantation Study in TAVI Patients (PORTRAIT-TAVI) is a retrospective, international, multicenter study that involved adult patients receiving a transcatheter bioprosthesis at 9 centers of cardiac surgery in Italy (5 centers), the Netherlands (1 center), Switzerland (1 center), Poland (1 center), and Germany (1 center) from July 2009 to January 2020. 

### 2.1. Ethical Statement

This study is registered in clinicaltrial.gov (Identifier: NCT03835598). The study was approved by the Institutional Review Board of each participating center. The need for individual patient consent for the study was waived by the committee. 

### 2.2. Inclusion and Exclusion Criteria

Patients older than 18 years who required a transcatheter biological aortic bioprosthesis for severe aortic stenosis were considered for the analysis. All patients were evaluated by a multidisciplinary heart team who determined TAVI indications, approach, and the type of transcatheter valves used. Patients were treated either with a balloon-expandable or a self-expandable valve. All patients gave written informed consent before the procedure. Patient demographics, symptoms, and comorbidities were documented, and individual risk was calculated by the logistic European System for Cardiac Operative Risk Evaluation (EuroSCORE). Transthoracic echocardiography was the initial screening examination used to evaluate the severity of aortic stenosis. TAVI access route and valve size were selected using computer tomography measurements.

Platelet counts were studied retrospectively, with available data analyzed from pre-implantation and on a daily basis (from day 0 to day 5) until discharge. Patients with a baseline platelet count below 150,000/µL were excluded from the analysis. Subjects with an oncologic disease, a concurrent infection or inflammatory disorder, or those who needed a concurrent percutaneous coronary intervention were also excluded from the study.

Following the procedure, all patients were given 300 mg of clopidogrel, and they were started on a double anti-aggregation treatment regimen that included 75 mg of clopidogrel and 100 mg of acetylsalicylic acid each day. Also, low-molecular-weight heparin was administered as a prophylactic deep vein thrombosis during the hospital stay.

Baseline characteristics, procedural data, and clinical outcomes were collected in a dedicated database after a robust check of its completeness and quality.

### 2.3. Definitions and Endpoints

The lowest recorded platelet count during hospitalization was defined as the nadir platelet count.

Thrombocytopenia occurs when platelet counts are less than 150 × 10^3^/µL. It is further classified as moderate (59–99 × 10^3^/µL), severe (<50 × 10^3^/µL), and mild (100–149 × 10^3^/µL). The adoption of a cut-off value of 100 × 10^3^/µL was considered the most appropriate to identify a pathologic condition related to thrombocytopenia [9,10]. The early adverse events associated with a platelet count below 100 × 10^3^/µL were investigated in the study population. Moreover, platelet count was analyzed by comparing SEV and BEV groups. The Valve Academic Research Consortium (VARC-3) criteria were used to define periprocedural events and mortality [11].

The primary aim of this study was to evaluate the frequency of the aforementioned outcomes in the overall population and the BEV and SEV groups. The secondary endpoint was to determine the risk factors for the development of periprocedural thrombocytopenia.

### 2.4. Statistical Analysis

Continuous variables are expressed as mean and standard deviation, or median and quartiles, respectively, for normally or non-normally distributed variables (as tested by the Shapiro–Wilk test) and were compared using Student’s *t*-test (or Wilcoxon–Mann–Whitney U test, as appropriate) and ANOVA (followed by the Tukey post hoc test) for multiple comparisons. Proportions are expressed as percentages and compared using the χ2 test or Fisher’s exact test, as appropriate.

Variables with a missing variable rate of more than 30% were excluded; otherwise, missing data were handled as follows: The mechanisms underlying missing data were investigated with sensitivity analysis and multiple imputation, generating five different datasets. The result of multiple treatment effects was pooled using the Rubin rules.

Propensity score matching was used to balance the distributions of the measured confounding baseline covariates between the SEV and BEV groups. The propensity score was obtained using logistic regression. Overlapping was assessed with common support plots. In addition, 1:1 matching was analyzed with different calipers ranging from 0.05 to 0.65, choosing the best one (0.20). The variables included in the propensity model are reported in Supplementary Figure S1.

The balance between the two matched groups was assessed with a standardized mean difference (SMD), considered optimal below 0.10. For the analysis of platelet counts over time, a mixed regression model was used, with time points as repeated measurements and patients as subjects. Logistic regression was used to assess the impact of the minimum post-procedural platelet count (<100.000/µL) on outcomes. 

The odds ratios (ORs) and 95% confidence limits were reported for both the unmatched and matched groups and adjusted for those variables showing SMD > 0.10. Generalized linear models with a logarithmic link function were used to evaluate the association between risk and the minimum post-procedural platelet count cut-off with the intensive care unit (ICU) and in-hospital length of stay (LoS).

Moreover, both ICU and in-hospital LoS were transformed into nominal variables using the median value as a cut-off, and then logistic regression was performed.

Univariate and multivariate analyses were performed to identify factors that might predict a platelet count value <100 × 10^3^/µL.

R-studio version 1.1.463 (2009–2018) was used for all statistical analyses. The significance of differences was considered at a *p* value < 0.05.

## 3. Results

### 3.1. Study Population

Supplementary Table S1 summarizes the baseline and procedural characteristics of the 1.122 patients enrolled in the study, comprising 395 patients (35.2%) who were treated with SEVs and 727 patients (64.8%) who received BEVs (Supplementary Table S1). Supplementary Tables S2–S4 show the results for the unmatched population. Propensity score matching yielded 316 patient pairs (Table 1). The average age of the overall matched population was 81 years old, and 83% had NYHA class III/IV symptoms at the time of the procedure. The mean aortic valve area index was 0.4 cm^2^/m^2^. Aspirin alone or dual antiplatelet therapy was taken by less than 4% of the overall population prior to valve implantation. Most TAVI procedures were elective, and the femoral artery was the preferred access route in both groups. 

### 3.2. Changes in Blood Elements after TAVI

**Figure 1:**
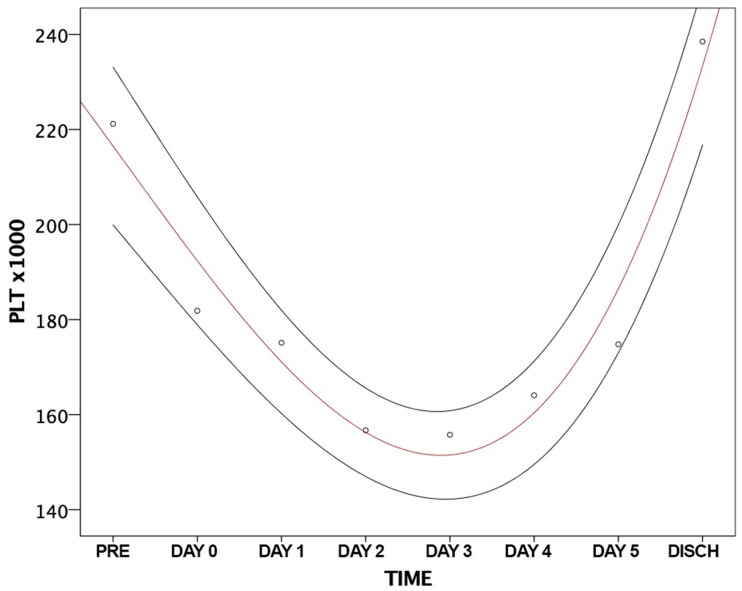
Platelet count variation after transcatheter aortic valve implantation. Abbreviation: PLT, platelet; Pre, pre-implantation; Disch, discharge.

Pre-operative platelet count was >150.000/µL in all patients, as per inclusion criteria. A mixed regression model for repeated-measure analysis showed that the post-procedural platelet count changed according to a parabolic curve in all patients (estimate = −0.931, standard error = 0.421, *p* = 0.027, Figure 1).

**Figure 2:**
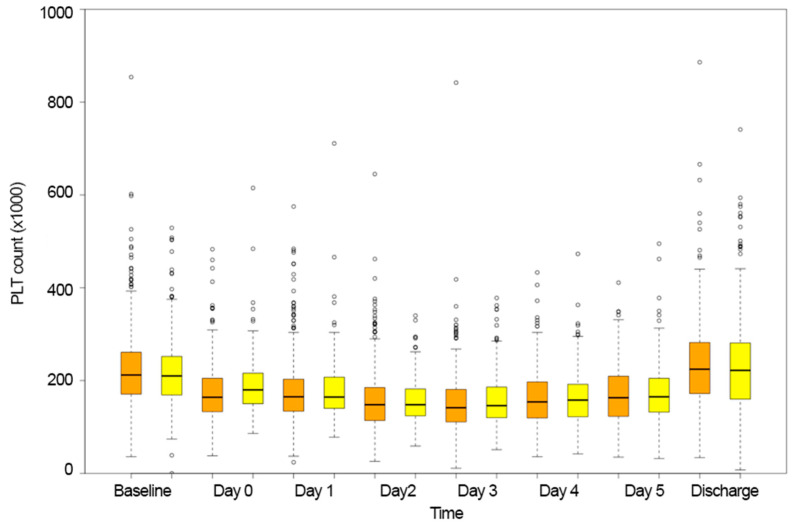
Platelet count variation over time in the balloon-expandable group (orange bars) and self-expandable valve group (yellow bars).

The platelet count varied similarly in both BEV and SEV patients (estimate = −4.276, standard error = 4.760, *p* = 0.369, Table 2, Figure 2). On average, the nadir platelet count value was recorded three days after implantation (BEV:146 (108–181) vs. SEV:149 (120–186), *p* = 0.142).

**Figure 3:**
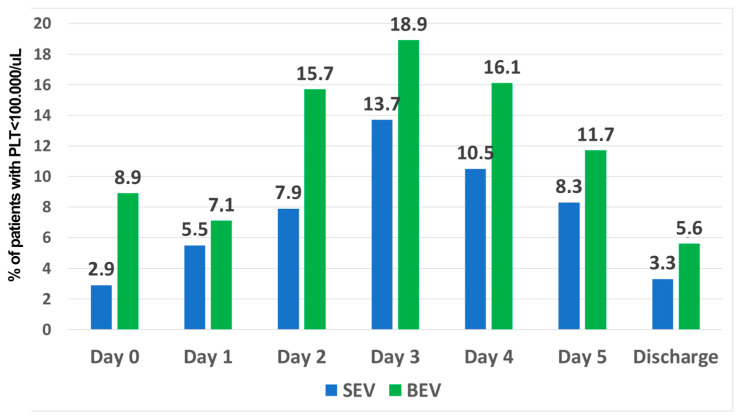
Rate of patients with thrombocytopenia in the balloon-expandable group (blue bars) and self-expandable valve group (green bars).

Post-procedural thrombocythemia was recorded in 14.6% of patients (92/632) without any difference between the two types of prostheses (BEV:51/316, SEV:41/316, *p* = 0.266, Figure 3). The RBC count and hemoglobin value decreased over time after TAVI implantation; however, no difference was found between the groups (Table 2).

An increased white blood cell (WBC) count was observed in the overall population, with the highest value between the 1st and the 2nd day after TAVI (Table 2). The SEV group showed the highest WBC value in the first three days after implantation, compared to the BEV group (Table 2).

### 3.3. Early Clinical Outcomes after TAVI

Nearly 22% of patients received RBC transfusions, but only 7% required more than two units (Table 3). Vascular problems and bleeding events affected 16% and 14% of the population, respectively (Table 3). The in-hospital mortality rate was similar between the groups (*p* = 0.102) and reached 6% in the overall population (Table 3). 

The platelet count <100 × 10^3^/µL was significantly associated with a higher need for blood product transfusions (RBC, *p* < 0.001; FFP, *p* = 0.007; PLT, *p* = 0.001, Table 4). Likewise, ICU LoS, in-hospital LoS, and in-hospital mortality were significantly associated with thrombocytopenia (Table 4). Given that the main objectives of this research were to investigate the impact of TAVI on the platelet count and identify the contributing factors to a drop below 100 × 10^3^/µL platelets, the results presented in Table 4 were corrected for variables with a statistically significant difference (SMD < 0.1) when matched, as previously mentioned in the Methods section.

Moreover, the univariate analyses revealed that a post-procedural platelet count <100 × 10^3^/µL was significantly associated with the male sex (OR: 1.69; *p* = 0.002); the 26 mm prosthesis size (OR: 1.667, *p* = 0.014); the 29 mm prosthesis size (OR: 1.81, *p* = 0.017); the baseline platelet count (OR: 0.972; *p*  < 0.001); prior liver cirrhosis (OR: 15.261, *p* = 0.012); and prior atrial fibrillation (OR: 2.132, *p* = 0.023) (Supplementary Table S5). Multivariate analysis showed that the BEV procedure (OR: 3.292, *p* = 0.045); dyslipidemia (OR:2.148, *p* = 0.048); the baseline platelet count (OR:0.975, *p* < 0.001); and prior liver cirrhosis (OR:12.109, *p* = 0.047) were predictors of a post-procedural platelet count <100 × 10^3^/µL (Supplementary Table S5).

## 4. Discussion

The main findings of the present international multicenter study revealed that (1) patients receiving TAVI were exposed to PR immediately after implantation; (2) PR occurred comparably in BEV and SEV patients; and (3) periprocedural thrombocytopenia was significantly associated with the need for RBC transfusions, prolonged ICU, and in-hospital LoS, as well as in-hospital mortality.

Several studies have shown that PR is a common phenomenon after both surgical and transcatheter aortic bioprosthesis implantation [5,12,13,14]. This phenomenon seems to be determined by the interaction of patients’ related factors and TAVI’s related factors. Even if we excluded patients with pre-operative thrombocytopenia, we found that the baseline platelet count value could be a predictor of a periprocedural low platelet count. Similarly, predisposing risk factors (such as liver cirrhosis) could increase the possibility of thrombocytopenia after TAVI. However, the procedure itself seems to be associated with a risk for platelet count decrease. Inflammatory reactions due to blood interaction with the artificial valve and mechanical platelet destruction due to shear stress modification have been considered to be concomitant causes of PR [5,6,15]. However, a few less credible hypotheses have been suggested to elucidate the phenomenon. Similar to the speculation advanced to explain PR after a stentless surgical bioprosthesis, a recent observational in vitro study analyzing platelet apoptosis biomarkers revealed that a formaldehyde-based storage solution of the prosthesis caused platelet injury [16,17]. However, the study was underpowered, and its results require further confirmation from large randomized trials. Thus, the use of iodinated contrast agents was aimed as another possible etiologic factor for PR [6,18].

While the underlying mechanisms are still to be clarified, mechanical platelet destruction could occur after the administration of hypo-osmolar iodinated contrast agents [18]. Several papers [6,8,19] described BEV patients as more vulnerable to PR because the delivery procedures typically required a large number of low-osmolar contrast agents to confirm appropriate valve positioning. However, this hypothesis was never confirmed by solid evidence. Furthermore, as physicians’ skills and technology for delivering prostheses improved over time, the need for a large number of iodinated contrast agents decreased, undermining this theory [20]. 

As a result, prosthesis design and delivery systems were believed to be important factors in PR. In the literature, several reports described the occurrence of PR more frequently after BEV implantation than after SEV implantation [7,8,13,17]. A recent systematic review and meta-analysis confirmed that more than 80% of patients receiving BEVs had thrombocytopenia, compared to almost 50% of patients undergoing SEV implantation [5]. Our multivariate analysis showed that BEV implantation was a significant predictor of a platelet count below 100 × 10^3^/µL. During BEV implantation, the use of the balloon could cause great endothelial damage and shear stress, triggering a higher decrease in the platelet count than in patients with SEVs [7]. Yet, this hypothesis is discordant with the different inflammatory responses elicited in BEV and SEV patients. We found a significantly increased WBC in SEV patients in the first three days after implantation. Abu Khadija and colleagues [21] reported a higher inflammatory response after SEV implantation than after the BEV procedure, which is consistent with our findings. The underlying explanation for the different immune responses could be ascribed to the distinct biocompatibility and materials between the prostheses (e.g., cobalt–chromium frame and bovine pericardial leaflets for BEVs vs. nitinol scaffold and porcine pericardial tissue for SEVs) [21]. Despite the different inflammatory responses, our results suggest that the platelet count varied regardless of the type of delivery system and prosthesis, contrasting what had previously been reported in smaller single-center retrospective studies [7,8,13,17]. Furthermore, Abu Khadija and colleagues confirmed that periprocedural thrombocytopenia was not associated with the SEV or BEV delivery systems, also comparing earlier and contemporary TAVI generations [21].

Beyond the numbers, platelet reduction is clinically relevant in TAVI patients. The association between the need for blood product transfusions, prolonged hospitalization, and in-hospital mortality is in line with other studies [7,8,21,22,23,24]. Hernandez-Enriquez and coauthors described a high rate of life-threatening bleeding events, major vascular complications, a greater need for RBC transfusions, and a high rate of sepsis and mortality in patients with a platelet count decrease over 30% at a 30-day follow-up [7]. Furthermore, Dvir and colleagues also reported prolonged ICU LoS and acute kidney injury as the drawbacks related to thrombocytopenia in TAVI patients [19]. Abu Khadija and associates reported that post-procedural PR was associated with higher rates of major bleeding, vascular complications, and mortality [21].

Zahid and colleagues found a greater incidence of bleeding complications associated with baseline hematological issues related to platelets and coagulation factors [25]. Significant bleeding episodes and blood problems are strictly correlated. It is important to emphasize the link between thrombocytopenia and life-threatening bleeding. Although there is a distinct correlation between these two phenomena, more research is necessary to fully understand the relationship. There are two potential outcomes: Either the patient experiences a bleeding episode followed by thrombocytopenia, or the patient experiences thrombocytopenia first and a hemorrhagic episode later. It is challenging to differentiate between the two situations because of the low rate of this event. Additional research in a prospective manner should be conducted to discriminate between the two possibilities.

However, taking into account the unfavorable effects of PR on clinical outcomes, TAVI implantation should be carefully examined in patients who are thrombocytopenic or who have a considerable risk of bleeding or other comorbidities. Moreover, currently, hospital stays are often shorter, and a drop in the platelet count might go unnoticed. A longer in-hospital length of stay should be considered for these patients in order to avoid overlooking a significant platelet drop with clinical impact.

### Study Limitations

Although the present study is the first to analyze platelet count variation in a large population of TAVI patients, it has limitations. First, due to its observational and retrospective nature, both selection bias and unmeasured confounders cannot be excluded. Second, due to the multicenter design, events were adjudicated by investigators at each center. Therefore, a certain degree of under-reporting of events cannot be completely ruled out. Furthermore, the prosthetics technology changed during the research period. Platelet decrease may have had a greater impact on early prosthetic generations than on more recent ones. Similar to this, in the early years of the study, multiple procedures were carried out using a trans-apical approach, but more recently, the femoral artery was the favored access route. There is a chance that the access route contributed to the platelet count’s lowering rates. 

In addition, biomarkers for platelet activation, inflammation, or hemolysis were not considered in this analysis. Lastly, heparin-induced thrombocytopenia was not investigated. However, heparin was found to have a limited role in post-TAVI thrombocytopenia [19,23,24].

## 5. Conclusions

Transcatheter aortic valve implantation is associated with a significant but transient PR, regardless of the type of prosthesis. TAVI patients who experience a PR below 100,000/µL are exposed to a high rate of early clinical adverse events and an increased in-hospital mortality rate. Prospective studies are needed to investigate and explain mechanisms and outcomes. 

## Figures and Tables

**Table 1:** Baseline and procedural characteristics of the matched study population according to the implanted valve.

	Overall Population (n = 632)	SEV (n = 316)	BEV (n = 316)	SMD
Age (years)	81.1 ± 6.1	81.3 ± 6.1)	80.9 ± 6.1)	0.07
Female gender	333 (52.7%)	171 (54.1%)	162 (51.3%)	0.06
BMI (kg/m^2^)	27.3 ± 4.9	27.3 ± 4.9	27.3 ± 4.9	−0.01
BSA (m^2^)	1.9 ± 0.2	1.8 ± 0.2	1.9 ± 0.2	−0.04
Hypertension	596 (94.3%)	299 (94.6%)	297 (94.0%)	0.06
Diabetes mellitus type II	249 (39.4%)	122 (38.6%)	127 (40.2%)	−0.04
Smoking	349 (55.2%)	172 (54.4%)	177 (56.0%)	−0.04
Dyslipidemia	460 (72.8%)	230 (72.8%)	230 (72.8%)	0.00
NYHA class				0.03
I	10 (1.6%)	5 (1.6%)	5 (1.6%)	
II	97 (15.3%)	52 (16.5%)	45 (14.2%)	
III	420 (66.5%)	209 (66.1%)	211 (66.8%)	
IV	105 (16.6%)	50 (15.8%)	55 (17.4%)	
COPD	128 (20.3%)	61 (19.3%)	67 (21.2%)	−0.06
Atrial fibrillation	238 (37.7%)	116 (36.7%)	122 (38.6%)	−0.04
Prior ischemic stroke	136 (21.5%)	84 (26.6%)	52 (16.5%)	0.33
PAD	93 (14.7%)	57 (18.0%)	36 (11.4%)	0.29
Prior MI	67 (10.6%)	33 (10.4%)	34 (10.8%)	−0.02
Prior PCI	172 (27.2%)	91 (28.8%)	81 (25.6%)	0.09
Prior CABG	112 (17.7%)	54 (17.1%)	58 (18.4%)	−0.01
Prior valve surgery	142 (22.5%)	69 (21.8%)	73 (23.1%)	−0.04
Prior ASA treatment	23 (3.6%)	12 (3.8%)	10 (3.2%)	0.10
Prior DAPT treatment	24 (3.8%)	14 (4.4%)	10 (3.2%)	0.15
Prior LMWH treatment	21 (3.3%)	12 (3.8%)	9 (2.8%)	0.17
Prior warfarin treatment	17 (2.7%)	9 (2.8%)	8 (2.5%)	0.06
Prior DOAC treatment	17 (2.7%)	10 (3.2%)	7 (2.2%)	0.16
EuroSCORE II	6.2 (3.8–10.6)	6.2 (4.1–11.8)	6.1 (3.6–11.0)	−0.09
Echocardiographic features				
AV peak gradient (mmHg)	62.9 ± 30.9	63.6 ± 30.9	62.2 ± 30.9	0.05
AV mean gradient (mmHg)	43.7 ± 14.2	43.9 ± 14.7	43.6 ± 13.8	0.01
AVA/BSA (cm^2^/m^2^)	0.40 ± 0.10	0.39 ± 0.09	0.39 ± 0.10	0.01
LVEF (%)	51.5 ± 12.8	51.3 ± 12.2	51.6 ± 13.5	−0.02
AR mean grade				0.06
None	297 (47.0%)	143 (45.3%)	154 (48.7%)	
Mild	72 (11.4%)	38 (12.0%)	34 (10.8%)	
Moderate	263 (41.6%)	135 (42.7%)	128 (40.5%)	
Severe	0 (0%)	0 (0%)	0 (0%)	
sPAP (mmHg)	53.4 ± 16.1	52.9 ± 16.3	53.9 ± 15.9	−0.06
Status				−0.08
Elective	586 (92.7%)	297 (94.0%)	289 (91.5%)	
Urgent	45 (7.1%)	18 (5.7%)	27 (8.5%)	
Emergent	1 (0.2%)	1 (0.3%)	0 (0%)	
Access				0.06
Femoral artery	474 (75.0%)	233 (73.7%)	241 (76.3%)	
LV apex	157 (24.8%)	83 (26.3%)	74 (23.4%)	
Ascending aorta	1 (0.2%)	0 (0%)	1 (0.3%)	
Carotid artery	0 (0%)	0 (0%)	0 (0%)	
Access conversion	6 (0.9%)	3 (0.9%)	3 (0.9%)	0.00

Abbreviations: SEV, self-expandable valve; BEV, balloon-expandable valve; SMD, standardized mean difference; BMI, body mass index; BSA, body surface area; NYHA, New York Heart Association; COPD, chronic obstructive pulmonary disease; PAD, peripheral artery disease, MI, myocardial infarction; PCI, percutaneous coronary intervention; CABG, coronary artery bypass grafting; ASA, aspirin or acetylsalicylic acid; DAPT, dual antiplatelet therapy; LMWH, low-molecular-weight heparin; DOAC, direct oral anticoagulant; AV, aortic valve; AVA, aortic valve area; LVEF, left ventricle ejection fraction; AR, aortic regurgitation; sPAP, systolic pulmonary artery pressure; LV, left ventricle. Age, BMI, BSA, AVA peak and mean gradient, AVA/BSA, LVEF, and sPAP are presented as mean ± standard deviation. EuroSCORE II is presented as median and interquartile range.

**Table 2:** Periprocedural laboratory values after transcatheter aortic implantation in the matched population.

TIME	Overall Population (n = 632)	SEV (n = 316)	BEV (n = 316)	*p*-Value
Platelet count (×10^3^ µL)				
Baseline	211 (171–254)	208 (169–252)	213 (177–261)	0.189
Day 0	175 (137–206)	176 (147–212)	164 (130–199)	0.216
Day 1	166 (137–205)	164 (138–208)	169 (137–202)	0.912
Day 2	149 (120–185)	150 (124–189)	149 (112–183)	0.330
Day 3	147 (114–186)	149 (120–186)	146 (108–181)	0.142
Day 4	155 (122–200)	162 (128–197)	147 (118–200)	0.203
Day 5	166 (126–212)	166 (136–207)	165 (122–218)	0.634
Discharge	227 (169–283)	234 (174–290)	217 (166–278)	0.196
Hemoglobin (g/dL)				
Baseline	12.3 (11.0–13.4)	12.3 (11.0–13.4)	12.2 (11.0–13.4)	0.945
Day 0	10.9 (9.9–12.1)	11.1 (10.0–12.1)	10.8 (9.9–12.2)	0.614
Day 1	10.4 (9.3–11.5)	10.5 (9.43–11.4)	10.4 (9.3–11.6)	0.810
Day 2	10.1 (9.1–11.3)	10.2 (9.1–11.3)	10.1 (9.1–11.2)	0.591
Day 3	9.9 (8.9–10.8)	10.1 (9.1–11.4)	9.9 (9.1–11.1)	0.147
Day 4	9.8 (9.0–11.1)	9.9 (9.0–11.2)	9.9 (9.0–11.2)	0.561
Day 5	9.5 (8.8–11.1)	10.1 (9.0–11.3)	10.0 (9.0–11.0)	0.124
Discharge	10.8 (9.67–11.7)	10.5 (9.5–11.7)	10.6 (9.5–11.7)	0.143
Red blood cells (×1,000,000/µL)				
Baseline	4.2 (3.8–4.6)	4.2 (3.8–4.6)	4.1 (3.7–4.5)	0.677
Day 0	3.7 (3.3–4.1)	3.8 (3.3–4.1)	3.7 (3.3–4.1)	0.638
Day 1	3.5 (3.2–3.9)	3.5 (3.1–3.9)	3.5 (3.2–3.9)	0.969
Day 2	3.5 (3.1–3.9)	3.4 (3.1–3.7)	3.4 (3.1–3.9)	0.623
Day 3	3.4 (3.1–3.8)	3.4 (3.1–3.8)	3.4 (3.1–3.8)	0.095
Day 4	3.4 (3.1–3.8)	3.4 (3.1–3.8)	3.4 (3.1–3.8)	0.335
Day 5	3.3 (3.1–3.7)	3.3 (3.0–3.8)	3.4 (3.1–3.7)	0.199
Discharge	3.6 (3.3–4.0)	3.6 (3.3–4.0)	3.6 (3.3–4.1)	0.493
White Blood cells (×10^3^ µL)				
Baseline	7.0 (5.8–8.4)	7.0 (5.8–8.2)	6.9 (5.8–8.7)	0.494
Day 0	8.5 (6.7–10.8)	8.5 (6.7–10.7)	8.6 (6.5–11.50)	0.503
Day 1	9.3 (7.7–11.5)	9.8 (7.9–12.3)	8.8 (7.4–10.9)	<0.001
Day 2	9.0 (7.3–11.4)	9.5 (7.6–12.1)	8.7 (7.2–10.3)	<0.001
Day 3	8.1 (6.6–10.1)	8.6 (6.8–10.5)	7.7 (6.4–9.4)	0.022
Day 4	7.7 (6.2–9.9)	8.0 (6.7–10.1)	7.5 (5.9–9.5)	0.188
Day 5	7.5 (6.2–9.6)	7.7 (6.0–9.7)	7.2 (5.8–9.0)	0.772
Discharge	7.2 (6.0–8.9)	7.3 (6.1–8.9)	7.1 (5.8–9.1)	0.192

Abbreviations: SEV, self-expandable valve; BEV, balloon-expandable valve. Variables are presented as median and interquartile range.

**Table 3:** Clinical outcomes after transcatheter aortic implantation in the matched population.

	Overall Population (n = 632)	SEV (n = 316)	BEV (n = 316)	*p*-Value
RBC transfusions				0.138
0	495 (78.3%)	256 (81.0%)	239 (75.6%)	
1	38 (6.0%)	9 (2.8%)	29 (9.2%)	
2	54 (8.5%)	28 (8.9%)	26 (8.2%)	
>2	45 (7.2%)	23 (7.2%)	22 (6.8%)	
Echocardiographic features				
AV peak gradient (mmHg)	21.6 ± 10.3	20.2 ± 10.7	23.7 ± 9.9	0.274
AV mean gradient (mmHg)	11.1 ± 5.5	10.1 ± 5.8	12.2 ± 5.3	0.662
LVEF (%)	55.4 ± 11.9	57.3 ± 11.6	53.9 ± 12.3	0.265
Intraprosthetic regurgitation	177 (28.1%)	102 (34.2%)	75 (24.9%)	0.065
Paravalvular leak	177 (28.1%)	75 (24.9%)	7 (34.2%)	0.065
Moderate or severe	27 (4.2%)	8 (2.5%)	19 (6.4%)	0.062
Antiplatelet/Anticoagulant therapy post-TAVI				
Post ASA treatment	338 (53.5%)	149 (47.2%)	189 (59.8%)	0.001
Post DAPT treatment	238 (37.7%)	125 (39.6%)	113 (35.8%)	0.384
Post LMWH treatment	12 (1.9%)	5 (1.6%)	7 (2.2%)	0.772
Post warfarin treatment	193 (31%)	96 (30.4%)	100 (31.6%)	0.731
Post DOAC treatment	41 (6.5%)	25 (7.9%)	16 (5.0%)	0.560
Early outcomes post-TAVI				
Intracranial bleeding	1 (0.2%)	0 (0%)	1 (0.3%)	1.000
Gastrointestinal bleeding	4 (0.6%)	2 (0.6%)	2 (0.6%)	1.000
Atrial fibrillation	30 (4.7%)	16 (6.1%)	14 (4.4%)	0.708
ICU LoS	1 (1–2)	1 (1–2)	1 (1–2)	0.211
In-hospital LoS	8 (7–13)	9 (7–13)	8 (7–12)	0.386
In-hospital mortality	40 (6.3%)	15 (4.7%)	25 (7.9%)	0.102

Abbreviations: SEV, self-expandable valve; BEV, balloon-expandable valve; RBC, red blood cell; ICU, intensive care unit; LoS, length of stay.

**Table 4:** The impact of minimum post-procedural platelet count (<100,000/µL) on outcomes.

	Predictive Estimate (95% Conf. Interval)	*p*-Value
Need for RBC transfusion	2.611 * (1.907–8.275)	<0.001
Need for FFP transfusion	23.022 * (2.533–209.216)	0.007
Need for PLT transfusion	21.941 * (4.474–107.612)	0.001
ICU LoS	1.907 ^†^ (1.537– 2.367)	<0.001
Prolonged ICU LoS ^‡^	1.851 * (1.147–2.988)	0.012
H LoS	1.086 ^†^ (0.948–1.246)	0.230
Prolonged H LoS ^§^	1.963 * (1.241–3.105)	0.004
In-hospital mortality	3.972 * (1.907–8.275)	<0.001

Abbreviations: RBC, red blood cell; FFP, fresh-frozen plasma; PLT, platelet; ICU, intensive care unit; LoS, length of stay, H LoS = in-hospital post-procedural length of stay; * odds ratio; ^†^ Exp(beta coefficient); ^‡^ prolonged ICU LoS: ICU LoS > 1st day; ^§^ prolonged H LoS: H LoS > 8th day.

## Data Availability

The data presented in this study are available upon request from the corresponding author.

## Supplementary Materials

The following supporting information can be downloaded at: https://www.mdpi.com/article/10.3390/jcm13061579/s1. Figure S1. Variables included in the propensity model. Table S1. Type of transcatheter prostheses. Table S2. Baseline and procedural characteristics of the unmatched study population according to the type of the implanted valve. Table S3. Periprocedural laboratory values after transcatheter aortic implantation in the unmatched population. Table S4. Clinical outcomes after transcatheter aortic implantation in the unmatched population. Table S5. Univariate and multivariate analyses.
