# Supplementary material for: Platelet Reduction after Transcatheter Aortic Valve Implantation: Results from the PORTRAIT Study
Source: J Clin Med. 2024 Mar 10;13(6):1579. doi: 10.3390/jcm13061579 (PMC10971425; doi:10.3390/jcm13061579)
Supplement: Supplementary file 1 [file jcm-13-01579-s001.zip › jcm-2884000-supplementary.pdf]

Platelet Reduction after Transcatheter Aortic Valve Implantation: Results from the PORTRAIT Study

Supplemental Material

Definition of patient characteristics

- **Hypertension:** according the 2020 guidelines by the International society of Hypertension. (Unger T, Borghi C, Charchar F, Khan NA, Poulter NR, Prabhakaran D, Ramirez A, Schlaich M, Stergiou GS, Tomaszewski M, Wainford RD, Williams B, Schutte AE. 2020 International Society of Hypertension Global Hypertension Practice Guidelines. Hypertension. 2020 Jun;75(6):1334-1357. doi: 10.1161/HYPERTENSIONAHA.120.15026.)
- **Diabetes Mellitus type II**: according the guidelines by the European Association for the study of Diabetes (Management of hyperglycaemia in type 2 diabetes, 2022. A consensus report by the American Diabetes Association (ADA) and the European Association for the Study of Diabetes (EASD) (2022) - https://www.easd.org/guidelines/statements-and-guidelines.html)
- **Smoking**: Tobacco smoking is the practice of burning tobacco with the smoke inhaled to be tasted and absorbed into the bloodstream. (Adams TN, Morris J. Smoking. [Updated 2023 May 22]. In: StatPearls [Internet]. Treasure Island (FL): StatPearls Publishing; 2024 Jan-. Available from: https://www.ncbi.nlm.nih.gov/books/NBK537066/)
- **Dyslipidemia**: it is the imbalance of lipids such as cholesterol, low-density lipoprotein cholesterol, (LDL-C), triglycerides, and high-density lipoprotein (HDL). (Pappan N, Rehman A. Dyslipidemia. [Updated 2023 Jul 10]. In: StatPearls [Internet]. Treasure Island (FL): StatPearls Publishing; 2024 Jan-. Available from: https://www.ncbi.nlm.nih.gov/books/NBK560891/)
- **NYHA class**: The NYHA classification is commonly used as a method for functional classification in patients with heart failure. (Raphael C, Briscoe C, Davies J, Ian Whinnett Z, Manisty C, Sutton R, Mayet J, Francis DP. Limitations of the New York Heart Association functional classification system and self-reported walking distances in chronic heart failure. Heart. 2007 Apr;93(4):476-82. doi: 10.1136/hrt.2006.089656.)
- **COPD**: a heterogeneous lung condition characterized by chronic respiratory symptoms (dyspnea, cough, expectoration, exacerbations) due to abnormalities of the airways (bronchitis, bronchiolitis) and/or alveoli (emphysema) that cause persistent, often progressive, airflow obstruction. (GLOBAL STRATEGY FOR PREVENTION, DIAGNOSIS AND MANAGEMENT OF COPD: 2023 Report https://goldcopd.org/2023-gold-report-2/)
- **Atrial Fibrillation**: A supraventricular tachyarrhythmia with uncoordinated atrial electrical activation and consequently ineffective atrial contraction. Electrocardiographic characteristics of AF include: Irregularly irregular R-R intervals (when atrioventricular conduction is not impaired); Absence of distinct repeating P waves, and Irregular atrial activations. (Gerhard Hindricks, et al, ESC Scientific Document Group , 2020 ESC Guidelines for the diagnosis and management of atrial fibrillation developed in collaboration with the European Association for Cardio-Thoracic Surgery (EACTS): The Task Force for the diagnosis and management of atrial fibrillation of the European Society of Cardiology (ESC) Developed with the special contribution of the European Heart Rhythm Association (EHRA) of the ESC, European Heart Journal, Volume 42, Issue 5, 1 February 2021, Pages 373–498, https://doi.org/10.1093/eurheartj/ehaa612)
- **Ischemic stroke**: defined by the AHA/ASA guidelines (Kleindorfer DO, et al. 2021 Guideline for the Prevention of Stroke in Patients With Stroke and Transient Ischemic Attack: A Guideline From the American Heart Association/American Stroke Association. Stroke. 2021 Jul;52(7):e364-e467. doi: 10.1161/STR.0000000000000375.)

- **Peripheral artery disease**: defined by the ESC and ESVS (Victor Aboyans, et al, ESC Scientific Document Group , 2017 ESC Guidelines on the Diagnosis and Treatment of Peripheral Arterial Diseases, in collaboration with the European Society for Vascular Surgery (ESVS): Document covering atherosclerotic disease of extracranial carotid and vertebral, mesenteric, renal, upper and lower extremity arteries Endorsed by: the European Stroke Organization (ESO); The Task Force for the Diagnosis and Treatment of Peripheral Arterial Diseases of the European Society of Cardiology (ESC) and of the European Society for Vascular Surgery (ESVS), European Heart Journal, Volume 39, Issue 9, 01 March 2018, Pages 763–816, https://doi.org/10.1093/eurheartj/ehx095
- **Myocardial infarction**: Fourth universal definition of myocardial infarction (2018) (Kristian Thygesen, et al, ESC Scientific Document Group , Fourth universal definition of myocardial infarction (2018), European Heart Journal, Volume 40, Issue 3, 14 January 2019, Pages 237–269, https://doi.org/10.1093/eurheartj/ehy462).

Figure S1. Variables included into the propensity model.

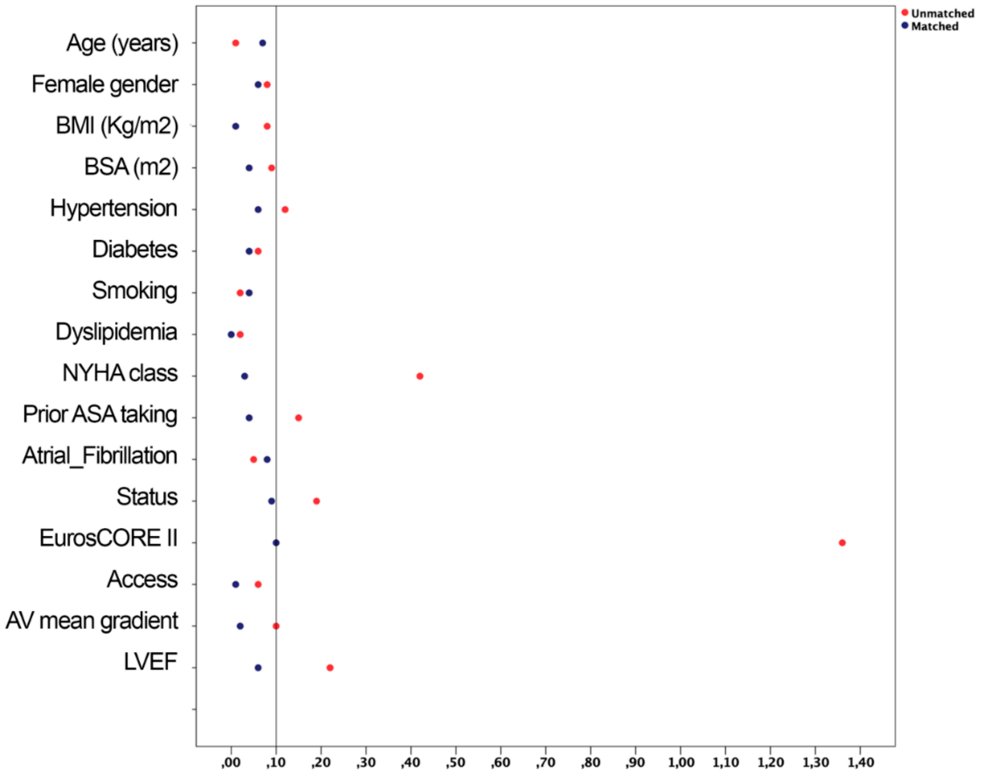

Table S1. Type of transcatheter prostheses.

	**Prosthesis type**	**Patients**
Balloon-expandable valve	Sapien	47
	Sapien XT	299
	Sapien 3	376
	Sapien Ultra	5
Self-expanding valve	CoreValve	73
	Evolut R	85
	Evolut R Pro	10
	Acurate	149

Acurate Neo	66
Engager	6
Portico	6

Table S2. Baseline and procedural characteristics of the unmatched study population according to the type of the implanted valve.

	Overall population (*n* = 1122)	SEV (*n* = 395)	BEV (*n* = 727)	SMD
Age (years)	81.3±6.0	81.3±6.0	81.3±6.1	0.01
Female gender	539 (48.0%)	180 (45.6%)	359 (49.4%)	-0.08
BMI (kg/m^2)	27.2±4.9	27.4±4.9	27.0±5.0	0.08
BSA (m^2)	1.84 (0.22)	1.83 (0.23)	1.85 (0.22)	-0.09
Hypertension	1.039 (92.6%)	362 (91.6%)	677 (93.1%)	-0.12
Diabetes Mellitus	429 (38.2%)	158 (40.0%)	271 (37.3%)	0.06
Smoking	530 (47.2%)	189 (47.8%)	341 (46.9%)	0.02
Dyslipidemia	778 (69.3%)	272 (68.9%)	506 (69.6%)	-0.02
NYHA class				-0.42
I	34 (3.0%)	27 (6.8%)	7 (1.0%)	
II	143 (12.7%)	81 (20.5%)	62 (8.5%)	
III	771 (68.8%)	236 (59.7%)	535 (73.6%)	
IV	174 (15.5%)	51 (12.9%)	123 (16.9%)	
COPD	267 (23.8%)	115 (29.1%)	152 (20.9%)	0.24
Atrial fibrillation	423 (37.7%)	133 (33.7%)	290 (39.9%)	-0.15
Prior ischemic stroke	197 (17.6%)	96 (24.3%)	101 (13.9%)	0.38
PAD	149 (13.3%)	66 (16.7%)	83 (11.4%)	0.24
Prior MI	130 (11.6%)	57 (14.4%)	73 (10.0%)	0.23
Prior PCI	305 (27.2%)	109 (27.6%)	196 (27.0%)	0.02
Prior CABG	193 (17.2%)	65 (16.5%)	128 (17.6%)	-0.04
Prior valve surgery	257 (22.9%)	77 (19.5%)	180 (24.8%)	-0.17
Prior ASA taking	67 (6.0%)	57 (14.4%)	10 (1.4%)	1.36
Prior DAPT taking	87 (7.8%)	77 (19.5%)	10 (1.4%)	1.56
Prior LMWH taking	35 (31.1%)	26 (6.6%)	9 (1.2%)	0.97
Prior warfarin taking	28 (2.5%)	15 (3.8%)	13 (1.8%)	0.42
Prior DOAC taking	21 (1.9%)	14 (3.5%)	7 (1.0%)	0.70
EuroSCORE II	6.1 (3.9-10.8)	5.6 (3.3-9.7)	6.4 (4.4-11.7)	0.19
Echocardiographic features				
AV peak gradient (mmHg)	67.2±32.9	66.6±32.1	67.5±33.4	-0.03
AV mean gradient (mmHg)	46.3±14.5	45.8±14.8	46.6±14.3	-0.06
AVA/BSA (cm^2/m^2)	0.38±0.09	0.39±0.09	0.38±0.09	0.11
LVEF (%)	52.3±12.9	51.4±11.7	52.7±13.6	-0.10
AR mean grade				0.04
None	529 (47.1%)	331 (45.5%)	198 (50.1%)	
Mild	147 (13.1%)	109 (15.0%)	38 (9.6%)	
Moderate	444 (39.6%)	287 (39.5%)	157 (39.7%)	
Severe	2 (0.2%)	0 (0%)	2 (0.5%)	
sPAP (mmHg)	54.5±15.5	55.5±15.1	52.3±16.1	0.20

**Status**				
Elective	1.036 (92.3%)	360 (91.1%)	676 (93.0%)	-0.14
Urgent	83 (7.4%)	33 (8.4%)	50 (6.9%)	0.12
Emergent	3 (0.3%)	2 (0.5%)	1 (0.1%)	-0.38
**Access**				-0.22
Femoral artery	791 (70.5%)	306 (77.5%)	485 (66.7%)	
LV apex	326 (29.1%)	87 (22.0%)	239 (32.9%)	
Ascending aorta	4 (0.4%)	1 (0.3%)	3 (0.4%)	
Carotid artery	1 (0.1%)	1 (0.3%)	0 (0%)	
**Access conversion**	14 (1.2%)	4 (1.0%)	10 (1.4%)	-0.19

Abbreviations: SEV, self-expanding valve; BEV, balloon expandable valve; SMD, standardized mean difference; BMI, body mass index; BSA, body surface area; NYHA, New York heart association; COPD, chronic obstructive pulmonary disease; PAD, peripheral artery disease; MI, myocardial infarction; PCI, percutaneous coronary intervention; CABG, coronary artery bypass grafting; ASA, aspirin or acetylsalicylic acid; DAPT, dual antiplatelet therapy; LMWH, low molecular weight heparin; DOAC, direct oral anticoagulant; AV, aortic valve; AVA, aortic valve area; LVEF, left ventricle ejection fraction; AR, aortic regurgitation; sPAPs, systolic pulmonary artery pressure; LV, left ventricle. Age, BMI, BSA, AVA peak and mean gradient, AVA/BSA, LVEF, and sPAP are presented as mean ± standard deviation. EuroSCORE II is presented as median and interquartile range.

Table S3. Peri-procedural laboratory values after transcatheter aortic implantation in the unmatched population.

TIME	Overall population (n = 1122)	SEV (n = 395)	BEV (n = 727)	p-value
**Platelet count (×1000/μL)**				
**Baseline**	212 (171-257)	210 (169-252)	212 (171-261)	0.280
**Day 0**	172 (141-213)	180 (150-216)	164 (132-205)	0.060
**Day 1**	165 (136-204)	165 (140-208)	165 (134-203)	0.739
**Day 2**	148 (117-184)	148 (124-183)	148 (114-185)	0.837
**Day 3**	144 (114-182)	146 (120-186)	142 (111-181)	0.326
**Day 4**	155 (120-195)	158 (122-192)	154 (119-197)	0.505
**Day 5**	164 (124-208)	165 (132-206)	163 (122-210)	0.285
**Discharge**	223 (169-282)	222 (160-281)	225 (171-283)	0.976
**Hemoglobin (g/dL)**				
**Baseline**	12.3 (11.0-13.5)	12.3 (11.0-13.3)	12.2 (10.9-13.3)	0.300
**Day 0**	11.2 (10.1-12.2)	10.8 (9.8-12.0)	10.7 (9.7-11.8)	0.070
**Day 1**	10.5 (9.3-11.5)	10.4 (9.4-11.4)	10.4 (9.4-11.4)	0.256
**Day 2**	9.9 (9.1-11.2)	10.1 (9.1-11.2)	10.1 (9.1-11.2)	0.896
**Day 3**	9.9 (9.0-10.9)	9.9 (9.0-11.1)	9.9 (9.1-11.1)	0.220
**Day 4**	9.7 (9.0-11.0)	9.8 (9.0-11.1)	9.9 (9.0-11.2)	0.451
**Day 5**	9.6 (8.8-11.0)	9.9 (9.0-11.0)	10.0 (9.0-11.0)	0.175
**Discharge**	10.4 (9.6-11.6)	10.5 (9.5-11.7)	10.6 (9.5-11.7)	0.518
**Red blood cells (×1,000,000/μL)**				
**Baseline**	4.1 (3.7-4.5)	4.2 (3.8-4.6)	4.1 (3.7-4.4)	0.300
**Day 0**	3.7 (3.3-4.0)	3.8 (3.4-4.1)	3.6 (3.3-3.9)	0.092
**Day 1**	3.5 (3.2-3.9)	3.6 (3.2-3.9)	3.5 (3.2-3.9)	0.165
**Day 2**	3.4 (3.1-3.8)	3.4 (3.1-3.9)	3.4 (3.1-3.8)	0.139
**Day 3**	3.4 (3.1-3.8)	3.4 (3.1-3.8)	3.4 (3.1-3.8)	0.715
**Day 4**	3.4 (3.1-3.8)	3.4 (3.1-3.8)	3.4 (3.1-3.8)	0.438
**Day 5**	3.4 (3.1-3.7)	3.3 (3.0-3.8)	3.4 (3.1-3.7)	0.494
**Discharge**	3.6 (3.2-4.0)	3.6 (3.2-4.0)	3.6 (3.3-4.0)	0.608
**White Blood cells (×1000/μL)**				

**Baseline**	6.9 (5.8-8.3)	7.1 (5.8-8.2)	6.9 (5.7-8.3)	0.293
**Day 0**	8.4 (6.8-10.7)	8.6 (6.9-11.0)	8.3 (6.7-10.5)	0.033
**Day 1**	9.3 (7.5-11.6)	9.8 (7.9-12.2)	9.1 (7.3-11.2)	0.005
**Day 2**	9.0 (7.2-11.5)	9.4 (7.6-12.2)	8.8 (7.1-11.1)	0.001
**Day 3**	8.1 (6.6-10.2)	8.6 (6.8-10.9)	7.8 (6.4-9.9)	0.082
**Day 4**	7.6 (6.2-9.9)	8.1 (6.7-10.2)	7.5 (6.0-9.6)	0.077
**Day 5**	7.4 (5.8-9.5)	7.6 (6.0-9.7)	7.2 (5.8-9.0)	0.613
**Discharge**	7.3 (6.0-9.1)	7.9 (6.4-10.1)	7.1 (5.8-8.7)	0.001

Abbreviations: SEV, self-expanding valve; BEV, balloon expandable valve. Variables are presented as median and interquartile range.

Table S4. Clinical outcomes after transcatheter aortic implantation in the unmatched population.

	**Overall population (*n* = 1122)**	**SEV (*n* = 395)**	**BEV (*n* = 727)**	**p-value**
**RBC transfused**				0.005
0	859 (76.6%)	568 (78.1%)	291 (73.7%)	
1	83 (7.4%)	34 (4.7%)	49 (12.4%)	
2	106 (9.4%)	74 (10.2%)	32 (8.1%)	
>2	74 (6.5%)	51 (7.0%)	23 (5.6%)	
**Bleeding**	183 (16.3%)	119 (16.4%)	64 (16.2%)	1.000
**Vascular complication**	156 (13.9%)	102 (14.0%)	54 (13.7%)	0.868
**Ischemic stroke**	25 (2.2%)	15 (2.1%)	10 (2.5%)	0.612
**Intracranial bleeding**	2 (0.2%)	1 (0.1%)	1 (0.3%)	1.000
**Gastrointestinal bleeding**	7 (0.7%)	4 (0.6%)	3 (0.8%)	0.702
**Atrial fibrillation**	61 (5.4%)	40 (5.5%)	21 (5.3%)	0.896
**ICU LoS**	1 (1-2)	1 (1-2)	1 (1-2)	0.697
**In-hospital LoS**	9 (7-13)	8 (7-13)	9 (7-13)	0.168
**In-hospital mortality**	66 (5.9%)	39 (5.4%)	27 (6.8%)	0.317

Abbreviations: SEV, self-expanding valve; BEV, balloon expandable valve; RBC, red blood cell; ICU, intensive care unit; LoS, length of stay.

Table S5. Univariate and multivariate analyses of factors associated with a post-procedural platelet count $<100\times10^3$/uL.

	Univariate				Multivariate			
	OR	95 CL		p-value	OR	95 CL		p-value
**Age**	1.012	0.985	1.041	0.382				
**Male**	1.69	1.22	2.36	0.002				
**TAVI: BEV**	1.43	0.999	2.03	0.055	3.292	1.827	1.014	0.045
**Hypertension**	0.807	0.419	1.555	0.522				
**Diabetes**	0.979	0.698	1.372	0.901				
**NYHA**	1.008	0.868	1.171	0.913				
**Smoking**	1.064	0.753	1.503	0.727				
**Dyslipidemia**	1.529	1.012	2.31	0.044	2.148	1.468	1.003	0.048
**LVEF**	0.996	0.989	1.003	0.219				
**AV Mean Gradient**	1.005	0.993	1.016	0.426				
**Transfemoral approach**	0.86	0.61	1.212	0.389				
**Size (vs 23)**								
**25**	0.84	0.38	1.857	0.666				
**26**	1.667	1.108	2.506	0.014				
**27**	1.619	0.824	3.179	0.162				
**29**	1.81	1.114	2.942	0.017				
**31**	2.32	0.881	6.11	0.088				
**34**	0.294	0.039	2.221	0.235				
**Baseline PLT count**	0.972	0.967	0.976	<0.001	0.975	0.971	0.966	<0.001
**Pre ASA taking**	0.72	0.338	1.534	0.394				
**COPD**	0.843	0.57	1.249	0.395				
**Liver cirrhosis**	15.261	4.606	1.39	0.012	12.109	3.512	1.019	0.047
**Dialysis**	2.402	0.95	0.376	0.914				
**Prior ischemic stroke**	1.71	1.081	0.684	0.739				
**PAD**	1.386	0.839	0.507	0.493				
**Previous MI**	1.229	0.693	0.391	0.21				
**Atrial Fibrillation**	2.132	1.502	1.058	0.023	1.996	1.409	0.994	0.054
**Pre Warfarin**	3.814	1.474	0.569	0.424				
**NOAC**	4.258	1.409	0,.67	0.543				
**Pre DAPT**	1.665	0.824	0.408	0.59				
**Urgent**	2.879	1.678	0.978	0.06				

Abbreviations: ASA, aspirin; AV, aortic valve; BEV, balloon expandable valve; COPD, chronic obstructive pulmonary disease; DAPT, dual antiplatelet therapy; LVEF, left ventricle ejection fraction; MI, myocardial infarction; NOAC, Non-vitamin K oral anticoagulants; NYHA, New York Heart Association; PAD, peripheral artery disease; PLT, platelet; TAVI, transcatheter aortic valve implantation.
